# Reshaping Dyslipidaemia Treatment with Bempedoic Acid—A Narrative Review

**DOI:** 10.3390/biomedicines13061460

**Published:** 2025-06-13

**Authors:** Dominik Strikic, Zvonimir Begic, Ivana Radman, Fran Zlopasa, Jana Mateljic, Ivica Zec, Marina Titlic, Ana Marija Sliskovic, Ivan Pecin, Zeljko Reiner, Iveta Mercep

**Affiliations:** 1 Division of Clinical Pharmacology, University Hospital Centre Zagreb, 10 000 Zagreb, Croatia; 2 Division of Plastic and Reconstructive Surgery, University Hospital Centre Zagreb, 10 000 Zagreb, Croatia; zvonimir.begic@hotmail.com; 3 Department of Ophthalmology, Sestre Milosrdnice University Hospital Centre, 10 000 Zagreb, Croatia; ivana.radman@gmail.com; 4 School of Medicine, University of Zagreb, 10 000 Zagreb, Croatia; fran.zlopasa13@gmail.com (F.Z.); jana.mateljic@gmail.com (J.M.);; 5 School of Medicine, University of Split, 21 000 Split, Croatia; marina.titlic@gmail.com; 6 Department of Cardiology, University Hospital Centre Zagreb, 10 000 Zagreb, Croatia; sliskovic_anamarija@yahoo.com; 7 Department of Internal Medicine, School of Medicine, University of Zagreb, 10 000 Zagreb, Croatia; ipecin@kbc-zagreb.hr (I.P.); imercep@gmail.com (I.M.); 8 Department of Cardiology and Congenital Diseases of Adults, Polish Mother’s Memorial Hospital Research Institute, 93-338 Lodz, Poland; zreiner@kbc-zagreb.hr

**Keywords:** dyslipidaemia, bempedoic acid, statin intolerance, cardiovascular risk, lipid-lowering therapy

## Abstract

Dyslipidaemia is one of the main causes of atherosclerotic cardiovascular disease (ASCVD) worldwide. Although statins remain the cornerstone of lipid-lowering therapy, many patients do not achieve optimal target levels of low-density lipoprotein cholesterol (LDL-C) due to intolerance or inadequate response. Bempedoic acid, an oral ATP citrate lyase inhibitor, provides a liver-specific mechanism that lowers LDL-C levels while minimising muscle-related side effects. Recent clinical trials, including the CLEAR Outcomes Study, have shown that bempedoic acid was able to reduce LDL-C by approximately 29 mg/dL and major adverse cardiovascular events (MACEs) by 13% in patients intolerant to statins. Combination therapy with ezetimibe further enhances this effect. However, adverse effects such as increased uric acid and gout have been reported, requiring careful patient selection and continuous monitoring. This review provides a comparative synthesis of the latest evidence on bempedoic acid, including its pharmacological profile, its efficacy in different patient groups, and its place within current treatment strategies for dyslipidaemia. It also identifies research gaps and directions for future studies.

## 1. Introduction

Dyslipidaemia is a major cause of cardiovascular disease (CVD) morbidity and mortality worldwide [1]. These disorders can be caused by genetic mutations, as in familial hypercholesterolaemia, or by secondary causes such as obesity, diabetes mellitus, and physical inactivity [2]. Elevated LDL cholesterol (LDL-C) has been shown to be causally linked to atherosclerosis and associated complications such as myocardial infarction, stroke, and peripheral arterial disease [3]. Globally, elevated LDL-C remains one of the leading risk factors for morbidity and mortality, contributing to millions of deaths and a significant number of disability-adjusted life years (DALYs) each year. In 2019 alone, high plasma LDL-C levels were responsible for approximately 4.4 million deaths and 98.6 million DALYs, highlighting the urgent need for effective treatment strategies [4].

Treatment of dyslipidaemia has traditionally relied on lifestyle modification and pharmacological interventions aimed at lowering LDL-C levels. Statins or HMG-CoA reductase inhibitors have been the cornerstone of dyslipidaemia treatment for decades. Their widespread use has significantly reduced the incidence of major adverse cardiovascular events (MACEs), including myocardial infarction, stroke, and cardiovascular mortality. However, despite their proven efficacy, a significant proportion of patients does not achieve optimal LDL-C targets due to statin intolerance or inadequate response to treatment. Common adverse effects such as myopathy and hepatotoxicity, as well as widespread misinformation about statins, often spread by the media and non-medical sources, have been shown to significantly reduce adherence to treatment. Studies report that up to 30% of patients discontinue statin therapy within the first year, often due to perceived side effects or fear influenced by non-evidence-based information [5,6]. Some patients suffer from statin intolerance, an adverse effect perceived by the patient as unpleasant or unacceptable, and/or certain laboratory abnormalities, both of which can be attributed to statin use [7]. Statin intolerance, defined by the National Lipid Association (NLA) as the inability to tolerate at least two different statins (one at the lowest starting dose), is reported in 5% to 30% of patients, although the true prevalence may be lower due to the nocebo effect [7,8]. For a syndrome to be classified as statin intolerance, symptoms must occur after initiation of therapy, improve after discontinuation of the statin, and recur when the statin is restarted. Some patients have partial intolerance, while others have complete intolerance [8,9]. In most cases, statin-associated muscle symptoms (SAMSs) are the reason for discontinuation of treatment. Muscle soreness, pain, cramps, fatigue, and/or weakness are reported as the most common SAMSs [7]. In some cases, statin therapy has been associated with myopathy, defined as unexplained muscle pain or weakness associated with an elevated creatine kinase (CK) level (>10 times the upper limit of normal), occurring in 1/10,000 patients per year. The rarest and most extreme form of SAMSs is rhabdomyolysis (CK > 40 times the upper limit of normal), which occurs in approximately 1/100,000 patients per year [7,9,10]. However, statins are a well-tolerated group of drugs.

Recent advances in the development of new lipid-lowering agents have produced new drugs that complement or offer alternatives to statins. Among these, proprotein convertase subtilisin/kexin type 9 (PCSK9) inhibitors, including monoclonal antibodies such as alirocumab and evolocumab, have revolutionised the field. These agents inhibit PCSK9, a protein that promotes the degradation of LDL receptors and thereby improves the clearance of LDL-C from the bloodstream. Clinical studies have shown that PCSK9 inhibitors can reduce LDL-C levels by 50–60%, significantly reducing the risk of MACE. Despite their proven efficacy, the need for frequent subcutaneous administration and the high cost of treatment have limited their widespread use [11].

Inclisiran, a small interfering RNA (siRNA) molecule that targets hepatic PCSK9 synthesis, offers a new and promising approach. By silencing PCSK9 production at the genetic level, Inclisiran provides a sustained reduction in LDL-C levels of up to 50% with a convenient six-monthly dosing schedule. This extended dosing interval has a positive impact on treatment adherence and represents a significant advance in the treatment of hypercholesterolaemia. Inclisiran has been shown to be effective in patients with statin intolerance and in patients who require combination therapy to achieve LDL-C targets, making it an important addition to the therapeutic armamentarium [12].

Bempedoic acid, a novel lipid-lowering agent, is characterised by its unique mechanism of action and its favourable tolerability profile. By inhibiting ATP citrate lyase, a key enzyme upstream of HMG-CoA reductase in the cholesterol biosynthesis pathway, bempedoic acid effectively lowers LDL-C levels [13]. The liver-specific activation of bempedoic acid minimises systemic side effects, especially those affecting the skeletal muscles. This property makes bempedoic acid a particularly attractive option for patients who cannot tolerate statins or who require additional LDL-C lowering despite maximising statin therapy [14]. Clinical studies have consistently shown that bempedoic acid not only achieves significant LDL-C lowering, but also shows promising results in terms of cardiovascular risk reduction, making it an important addition to the treatment paradigm for dyslipidaemia [15,16].

This review aims to provide a comprehensive and updated analysis of bempedoic acid, incorporating recent data from clinical trials, including metabolic and gender subgroup analyses. It critically evaluates the efficacy, safety, and positioning of this agent in comparison to other contemporary non-statin therapies.

This review sets itself apart from other studies by incorporating newly published results from the CLEAR Outcomes and CLEAR Harmony trials up to the end of 2024, including data on metabolic safety in different glycaemic subgroups, real-world efficacy, and gender-specific outcomes. In addition, bempedoic acid is compared with other emerging non-statin therapies, providing a more practical perspective for clinical decision-making in patients with statin intolerance.

## 2. Methods

A structured literature search of PubMed, Scopus, Embase, and Web of Science was contucted between 1 June 2024 and 31 December 2024. The search strategy included combinations of the following MeSH terms and keywords using Boolean operators (AND/OR): “bempedoic acid”, “ETC-1002”, “LDL-C”, “statin intolerance”, “cardiovascular outcomes”, “safety”, and “efficacy”. Only English-language, peer-reviewed human studies published from January 2019 to December 2024 were included. Reviews, randomised controlled trials (RCTs), and real-world observational studies were eligible. Articles were screened manually for relevance. Risk of bias was not formally assessed due to the narrative nature of this review, but preference was given to large, well-conducted trials and meta-analyses.

## 3. Bempedoic Acid

### 3.1. Pharmacokinetics

Statins are widely used drugs to lower elevated plasma LDL-C levels, whether they are used as monotherapy or together with ezetimibe. Nevertheless, the high percentage of statin intolerance and/or adverse muscle-related side effects has stimulated research to find alternative drugs to lower elevated LDL-C levels [17]. Bempedoic acid is an oral medication that was developed in 2003 and is used to lower LDL-C levels. Due to its unique molecular structure, bempedoic acid is an oral prodrug that requires hepatic activation by very long-chain acyl-CoA synthetase 1 (ACSVL1), an enzyme that is expressed exclusively in the liver and is not found in skeletal muscle, significantly reducing the risk of myotoxicity [18].

This liver-specific activation is supported by pharmacokinetic data showing that the drug is no longer detectable in the systemic circulation within 24 h of administration [19]. Following absorption, the prodrug is converted to its active CoA form only in hepatocytes, minimising off-target exposure and contributing to its favourable safety profile [19,20]. Bempedoic acid is available in the form of 180 mg tablets and is characterised by good gastrointestinal bioavailability and tolerability. Considering the terminal half-life of approximately 21 h and ~2.3-fold accumulation at steady state, optimal efficacy is achieved with once-daily dosing [20]. Bempedoic acid exhibits good plasma protein binding, with approximately 99% being bound to plasma proteins, mainly albumin [21]. The drug undergoes extensive metabolism by glucuronidation, mainly via UGT2B7, and oxidative conversion to keto-metabolites, including ESP15228 [22,23].

Bempedoic acid is excreted primarily via urine and faeces, with approximately 70% of the administered dose being recovered via faeces and approximately 30% via urine [24].

### 3.2. Pharmacodynamics

**Figure 1:**
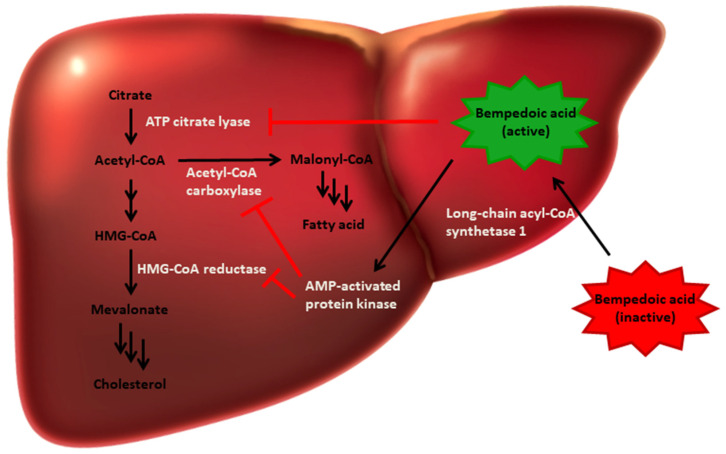
Mechanism of action of bempedoic acid. The drug inhibits ATP citrate lyase (ACLY), reducing the availability of acetyl-CoA for cholesterol and fatty acid synthesis, and activates AMPK, downregulating lipogenic enzymes such as HMG-CoA reductase and acetyl-CoA carboxylase.

It is a dicarboxylic acid derivative that acts both as an activator of hepatic adenosine monophosphate-activated protein kinase (AMPK) and as an inhibitor of ATP citrate lyase (ACLY). Through these mechanisms, bempedoic acid interrupts sterol and fatty acid synthesis at several points in the lipid biosynthetic pathway. AMPK activation leads to inhibitory phosphorylation of key enzymes such as acetyl-CoA carboxylase and HMG-CoA reductase, which are involved in fatty acid synthesis. ACLY plays a central role in linking glucose metabolism and lipogenesis by catalysing the cleavage of mitochondrial citrate into cytosolic acetyl-CoA and oxaloacetate. Inhibition of ACLY reduces the availability of acetyl-CoA in the cytosol and thus restricts the downstream synthesis of fatty acids and sterols [19,25,26] (Figure 1).

In cases where a stronger cholesterol-lowering effect is required, bempedoic acid can be taken together with ezetimibe as separate tablets or as a fixed-dose combination. The cholesterol-lowering effect of ezetimibe is based on the inhibition of the activity of the Niemann-Pick C1 like 1 (NPC1L1) protein. This protein acts as a sterol transporter in the intestine, and its inhibition reduces the absorption of cholesterol into the blood [27]. Ezetimibe is the drug of choice when patients fail to lower LDL-C levels sufficiently to reach target levels or when statins have too many adverse effects. As a rule, 10 mg ezetimibe is added to the combination therapy [28,29,30].

### 3.3. Efficacy

Bempedoic acid has been extensively studied in several clinical studies. In an early phase 2 double-blind study in patients with hypercholesterolaemia and statin intolerance (statin-associated muscle discomfort), bempedoic acid showed significant results in reducing LDL-C. The dose of bempedoic acid was increased in 2-week intervals from 60 mg daily to a maximum dose of 240 mg. Bempedoic acid was administered for a total of 8 weeks. At week 8, the mean LDL-C level decreased from 162 mg/dL to 115 mg/dL in the bempedoic acid group, representing a 28.7% reduction, compared to a 3.3% reduction in the placebo group. Non-HDL-C was reduced by 25.4% (213 mg/dL to 157 mg/dL) in the control group, compared to a 4.4% reduction in the placebo group. There were also significant differences between the two groups in other parameters. In the bempedoic acid group, total cholesterol was decreased by 18.4%, apolipoprotein B by 15.3%, and hsCRP by 42% [31].

In the phase 2b extension of the study, which evaluated the effects of bempedoic acid both as monotherapy and in combination with ezetimibe on LDL-C levels, significant reductions were observed across patient groups, regardless of statin intolerance status. The treatment period in this study was 12 weeks, and the largest LDL-C reductions of 43% and 48% were seen in patients receiving the combination of bempedoic acid 120 mg or 180 mg with ezetimibe. Monotherapy with bempedoic acid 120 mg or 180 mg lowered LDL-C levels by 28% and 30%. No significant difference was found between statin-intolerant and statin-tolerant patients. Apolipoprotein B, total cholesterol, and non-HDL-C were also reduced, particularly in the combination therapy, while hsCRP was reduced particularly in the monotherapy group with 180 mg bempedoic acid (40%) [32]. In both phase 2 studies, significant results were achieved after only 2 weeks of taking the drug [31,32]. Similar results were also observed in the later phase 3 clinical trial [33,34].

The CLEAR Tranquilly study involved patients with an LDL-C level of at least 70 mg/dL who took the maximum dose of a statin with or without an additional drug. After 12 weeks, the difference in LDL-C levels decreased by 23.5% (from 129.8 mg/dL at baseline to 96.2 mg/dL at week 12) in the group treated with bempedoic acid compared to the placebo group. Other parameters such as non-HDL cholesterol, total cholesterol, apoB protein, and hsCRP were also more than 10% lower in the groups taking bempedoic acid, with the difference in hsCRP being the greatest at 32.5%. The efficacy of the drug did not vary depending upon the intake of other lipid-lowering drugs. A greater reduction in LDL-C was observed in female participants compared to male participants, with mean decreases of 22.3% and 17.4%, respectively, suggesting a possible sex-based difference in treatment response [19,34].

In phase 3 of the CLEAR Serenity study, patients who could not tolerate statins were treated with 180 mg bempedoic acid per day for 24 weeks. After 12 weeks, a 21.4% reduction in LDL-C levels was observed in the bempedoic acid-treated group compared to the placebo group (−39.3 mg/dL compared to −3.1 mg/dL). As in previous studies, a significant decrease in non-HDL-C, total cholesterol, hsCRP, and apoB was also observed in this study compared to the placebo group. In contrast to other parameters, hsCRP continued to decrease significantly between 12 and 24 weeks. In the post hoc analysis, the patients who had no lipid-modifying background therapy (−22.1%) or a non-statin background therapy (−23.3%), in contrast to the group who had a background therapy with a very low dose statin (−17.4%), showed a greater reduction in LDL-C levels with the bempedoic acid compared to the placebo group [20].

A recent double-blind, placebo-controlled study on 13,970 statin-intolerant patients at high cardiovascular risk showed that bempedoic acid significantly lowered LDL-C levels and reduced the incidence of MACE. At a median follow-up of 40.6 months, bempedoic acid lowered LDL-C levels by 29.2 mg/dL and was associated with a 13% relative reduction in the risk of MACE compared to the placebo group, including AMI and coronary revascularisation. While the treatment was generally well-tolerated, a slightly increased incidence of gout and cholelithiasis was noted compared to the placebo group (3.1% vs. 2.1% for gout; 2.2% vs. 1.2% for cholelithiasis). These results emphasise that bempedoic acid is an effective alternative for patients who cannot tolerate statins [35,36].

The CLEAR Outcomes trial, a randomised, double-blind, placebo-controlled study involving 13,970 statin-intolerant patients at high cardiovascular risk, provided detailed insights into the efficacy and safety of bempedoic acid across different glycemic statuses. Bempedoic acid led to a significant placebo-corrected reduction in LDL-C levels across all glycemic strata. At 6 months, the mean LDL-C decreased by 21.1% compared to placebo (139 mg/dL to 107 mg/dL in bempedoic acid group vs. 139 mg/dL to 136 mg/dL in the placebo group). hsCRP, a marker of inflammation, was reduced by 21.6% in the bempedoic acid group compared to placebo after 6 months. Among patients with diabetes, bempedoic acid reduced the risk of (MACE) by 17% compared to placebo with an absolute risk reduction of 2.4%. Importantly, bempedoic acid did not increase the risk of new-onset diabetes. The incidence was 11.1% in the bempedoic acid group versus 11.5% in the placebo group (HR 0.95; 95% CI 0.83–1.09). Additionally, there were no significant changes in HbA1c levels among patients without diabetes at baseline. These results emphasise that bempedoic acid is an effective and metabolically safe alternative for the management of cardiovascular risk in patients with or without diabetes [35].

In addition to data from controlled clinical trials, real-world evidence also supports the efficacy and safety of bempedoic acid in routine clinical practise. Observational data and systematic reviews suggest that bempedoic acid consistently lowers LDL-C by 17–25%, even in patients who are intolerant to statins or who are already receiving lipid-lowering therapy [36]. A large meta-analysis has shown that bempedoic acid reduces the number of major adverse cardiovascular events (MACEs), particularly non-fatal myocardial infarctions and coronary revascularisations, with a favourable number of treatments required [37,38,39]. In addition, it is clear in practise that the drug has minimal muscle-related side effects due to its liver-selective activation, making it a valuable alternative for patients who cannot tolerate statins [40].

### 3.4. Adverse Effects

The safety profile of bempedoic acid has been thoroughly investigated in numerous clinical studies, including both randomised controlled studies and real-world data. In the published studies on bempedoic acid, most adverse reactions occurred with similar frequency in the drug-treated groups as in the placebo groups. The most common adverse effects included hyperuricaemia, gout, increased liver enzyme activity, muscle cramps, and pain in the extremities. An increased incidence of hyperuricaemia and gout is thought to be due to the inhibition of renal organic anion transporter 2 (OAT2), which plays a role in the excretion of uric acid. This mechanism is supported by preclinical models showing that bempedoic acid inhibits OAT2-mediated uric acid transport [35,41]. In the CLEAR Outcomes study, which included patients with statin intolerance, the overall incidence of adverse effects was 86.3% in patients treated with bempedoic acid compared to 85% in the placebo group, and serious adverse effects were observed in 25% of patients in both groups. Impaired renal function occurred in 11.5% of patients treated with bempedoic acid compared to 8.5% in the placebo group, and elevations in liver enzymes were also slightly more common. In the same study, the number of gout cases (NNH) was approximately 37 over a median follow-up of 40.6 months, which corresponds to an incidence rate of about 9 cases per 1000 patient-years.

In the CLEAR Harmony trial, 10.9% of patients in the bempedoic acid group versus 7.1% in the placebo group discontinued treatment due to adverse events. In contrast, in the CLEAR Outcomes study, the rates of treatment discontinuation were similar in the two groups—11.3% in the bempedoic acid group and 11.2% in the placebogroup—indicating no significant difference [19,35,42].

In addition, the safety profile of bempedoic acid in the CLEAR Harmony study was comparable to placebo, with no significant differences in overall adverse events. However, a higher incidence of gout and discontinuations was noted in the bempedoic acid group [19].

Tendon ruptures were also reported as a rare but notable adverse effect. They occurred in approximately 0.5% of patients treated with bempedoic acid and most involved the Achilles tendon, biceps tendon, or rotator cuff. This was more common in patients with risk factors such as advanced age, corticosteroid use, or concomitant statin therapy [36].

Overall, bempedoic acid has a safety profile comparable to placebo and a similar incidence of adverse events. While most adverse events occurred with comparable frequency in the drug-treated group and the placebo group, a higher incidence of gout, renal dysfunction, and discontinuations due to adverse effects was observed in the bempedoic acid group. The antagonistic effect of bempedoic acid on the renal OAT-2 transporter may contribute to these results. In studies such as CLEAR Outcomes and CLEAR Harmony, the overall incidence of adverse events was high, although serious events and discontinuations were comparable to placebo [43] (Table 1).

## 4. Discussion

Bempedoic acid is an oral medication for the treatment of elevated LDL-C levels and is an alternative for patients who cannot tolerate statins due to muscle-related adverse effects. Its unique pharmacological profile involves the inhibition of hepatic ATP citrate lyase (ACLY) and the activation of adenosine monophosphate-activated protein kinase (AMPK), which act synergistically to reduce cholesterol synthesis in the liver. By selectively acting on the liver, bempedoic acid minimises off-target effects on skeletal muscle, improving its safety profile [19].

Clinical studies, including the CLEAR Outcomes and CLEAR Harmony studies, have demonstrated the efficacy of bempedoic acid in reducing LDL-C levels and associated cardiovascular events. In the CLEAR Outcomes study, which involved statin-intolerant patients, bempedoic acid significantly reduced LDL-C levels and MACE with a median follow-up of 40.6 months. Although the overall safety profile of bempedoic acid was consistent with the placebo, a higher incidence of gout, renal dysfunction, and discontinuations due to adverse effects was observed [44,45,46,47].

The European Medicines Agency (EMA) has granted marketing authorisation for bempedoic acid, recognising its potential as an effective alternative for treating high LDL-C levels and reducing cardiovascular risk in patients with statin intolerance [48]. According to the EMA’s European Public Assessment Report (EPAR), bempedoic acid has been evaluated in clinical trials that demonstrated its benefits in lowering LDL-C levels while maintaining a favourable safety profile. Although side events were generally comparable to those of the placebo, an increased risk of gout and impaired renal function was observed, which is attributed to the drug’s antagonistic effect on the renal OAT-2 transporter.

The CLEAR Harmony study confirmed these findings and showed that the safety profile of bempedoic acid was comparable to the placebo, but with a higher rate of gout and increased discontinuations of treatment. The EMA recognises that despite these risks, bempedoic acid is an important therapeutic option for patients who cannot tolerate statins, particularly given its proven efficacy in reducing LDL-C levels and cardiovascular risk. However, EMA advises monitoring serum uric acid levels at regular intervals when clinically indicated. If hyperuricaemia is associated with gout symptoms, treatment with bempedoic acid should be discontinued [48].

## 5. Conclusions

Bempedoic acid has proven to be a promising LDL-C-lowering agent and is an important alternative for patients who cannot tolerate statins or who require an additional reduction in LDL-C levels despite intensive statin therapy. Its unique mechanism of action combined with a favourable safety profile makes it a valuable addition to the therapeutic armamentarium for treating dyslipidaemia and reducing cardiovascular risk. Clinical studies have consistently demonstrated the efficacy of bempedoic acid in lowering LDL-C levels and its potential to reduce serious cardiovascular events. Whilst adverse effects such as gout and renal dysfunction have been noted, they appear to be manageable with appropriate monitoring and clinical approach. However, some questions remain that require further investigation. The long-term effects of bempedoic acid on cardiovascular health beyond the observed study periods require more extensive longitudinal studies. In addition, understanding the full spectrum of metabolic effects, particularly in populations with different comorbidities and genetic backgrounds, is important to ensure broad applicability of this drug. In addition, the development of strategies to minimise side effects such as gout and kidney damage could improve overall tolerability of this drug and patient compliance.

In summary, the overall positive effects of bempedoic acid on lowering LDL-C levels and reducing the incidence of cardiovascular death position this novel drug at the forefront of the treatment of dyslipidaemia, particularly in individuals who cannot tolerate the statin treatment.

## Figures and Tables

**Table 1:** Summary of randomised clinical trials evaluating bempedoic acid.

Phase	Design	Sample Size	Population	Dose	LDL-C Reduction	Main Adverse Events
Thompson et al. (2015) [31]						
Phase 2	Randomized, double-blind, placebo-controlled	56	Statin-intolerant patients	ETC-1002 60–240 mg	−28.7% vs. placebo	Generally mild muscle effects, no rhabdomyolysis
Thompson et al. (2016) [32]						
Phase 2b	Randomized, double-blind, active comparator-controlled	349	Hypercholesterolemia ± statin intolerance	ETC-1002 120 or 180 mg ± ezetimibe 10 mg	~30% mono/~43–48% combo	Well-tolerated, no serious muscle symptoms
CLEAR Tranquility (2018) [34]						
Phase 3	Randomized, double-blind, placebo-controlled	269 (181 BA, 88 placebo)	Statin-intolerant patients on ezetimibe	BA 180 mg + EZE 10 mg	−28.5% vs. placebo	Muscle effects, blood uric acid increased, headache, urinary tract infection
CLEAR Serenity (2019) [25]						
Phase 3	Randomized, double-blind, placebo-controlled	345 (230 BA, 115 placebo)	Statin-intolerant patients	BA 180 mg	−21.4% vs. placebo	CV events (3.8%)
CLEAR Harmony (2019) [18]						
Phase 3	Randomized, double-blind, placebo-controlled	2230 (1488 BA, 742 placebo)	Patients with ASCVD or HeFH on max statin therapy	BA 180 mg	−16.5% vs. placebo	Muscle effects, upper respiratory tract infections
CLEAR Wisdom (2019) [43]						
Phase 3	Randomized, double-blind, placebo-controlled	779 (520 BA, 260 placebo)	Patients with ASCVD or HeFH on max statin therapy	BA 180 mg	15.1% less vs. placebo	Nasopharyngitis, UTI, hyperuricemia
Ballantyne et al. (2020) [33]						
Phase 3	Randomized, double-blind, placebo-controlled	301 (86 BA + EZE, 88 BA, 86 EZE, 41 placebo)	Patients with ASCVD, HeFH or CV risk factors	BA 180 mg + EZE 10 mg	−36.2% combo (placebo +1.8%, placebo corrected difference −38%), BA −17.2%, EZE −23.2%	Tolerability similar across all arms; UTI, nasopharyngitis, constipation, back pain, fatigue, upper respiratory tract infection, blood creatinine increased, bronchitis, headache, arthralgia, acute myocardial infarction, muscle spasms, myalgia
Laufs et al. (2019) [20]						
Phase 3	Randomized, double-blind, placebo-controlled, parallel group	345 statin-free patients (234 BA, 111 placebo)	Patients with hypercholesterolemia not receiving statins	BA 180 mg	−21.4% vs. placebo (placebo corrected difference) at 12 weeks	Similar AE frequency; muscle-related less common than in placebo group: myalgia (4.7%), pain in extremity (5.6%), muscle spasms (4.3%), arthralgia (6%), hypertension (4.3%)
Nissen et al. (2023)—CLEAR Outcomes [36]						
Phase 3	Randomized, double-blind, placebo-controlled	13,970 (6992 BA, 6978 placebo)	Statin-intolerant patients with ASCVD or high CV risk, age 15–85	BA 180 mg	−21.1% vs. placebo −0.8%, difference −20.3%	Gout (3.1%), cholelithiasis (2.2%), hyperuricemia (10.9%), renal impairment (11.5%)
